# An accelerated proximal augmented Lagrangian method and its application in compressive sensing

**DOI:** 10.1186/s13660-017-1539-0

**Published:** 2017-10-23

**Authors:** Min Sun, Jing Liu

**Affiliations:** 1 School of Management, Qufu Normal University, Shandong, 276826 P.R. China; 2 School of Mathematics and Statistics, Zaozhuang University, Shandong, 277160 P.R. China; 3 School of Data Sciences, Zhejiang University of Finance and Economics, Zhejiang, 310018 P.R. China

**Keywords:** 90C25, 90C30, proximal augmented Lagrangian multiplier method, convex programming, global convergence, compressive sensing

## Abstract

As a first-order method, the augmented Lagrangian method (ALM) is a benchmark solver for linearly constrained convex programming, and in practice some semi-definite proximal terms are often added to its primal variable’s subproblem to make it more implementable. In this paper, we propose an accelerated PALM with indefinite proximal regularization (PALM-IPR) for convex programming with linear constraints, which generalizes the proximal terms from semi-definite to indefinite. Under mild assumptions, we establish the worst-case $\mathcal{O}(1/t^{2})$ convergence rate of PALM-IPR in a non-ergodic sense. Finally, numerical results show that our new method is feasible and efficient for solving compressive sensing.

## Introduction

Let $\mathcal{R}$ denote the set of all real numbers, $\mathcal{R} ^{n}$ the Euclidean space of all real vectors with *n* coordinates. In this paper, we are going to solve the following linearly constrained convex programming:

1 $$ \min \bigl\{ f(x)|Ax=b, x\in\mathcal{R}^{n} \bigr\} , $$

where $f(x): \mathcal{R}^{n}\rightarrow\mathcal{R}$ is a closed proper convex function, $A\in\mathcal{R}^{m\times n}$, $b\in\mathcal{R}^{m}$. Throughout, we assume that the solution set of Problem (1) is nonempty. By choosing different objective function $f(x)$, a variety of problems encountered in compressive processing, machine learning and statistics can be cast into Problem (1) (see [1–8] and the references therein). The following are two concrete examples of Problem (1):

• The compressive sensing (CS):

2 $$ \begin{aligned} &\min_{x} f(x)= \Vert x \Vert _{1}+\frac{1}{2\mu} \Vert x \Vert _{2}^{2}, \\ &\textrm{s.t. } Ax=b, \end{aligned} $$

where $\mu>0, A\in\mathcal{R}^{m\times n}(m\ll n)$ is the sensing matrix; $b\in\mathcal{R}^{m}$ is the observed signal, and the $\ell_{1}$-norm and $\ell_{2}$-norm of the vector *x* are defined by $\Vert x \Vert _{1}=\sum_{i=1}^{n}\vert x_{i} \vert $ and $\Vert x \Vert _{2} = (\sum_{i=1}^{n}x_{i}^{2} )^{1/2}$, respectively.

• The wavelet-based image processing problem:

3 $$ \begin{aligned} &\min_{x} f(x)= \Vert x \Vert _{1}, \\ &\textrm{s.t. } BWx=b, \end{aligned} $$

where $B\in\mathcal{R}^{m\times l}$ is a diagonal matrix whose elements are either 0 (missing pixels) or 1 (known pixels), and $W\in \mathcal{R}^{l\times n}$ is a wavelet dictionary.

Problem (1) can be converted into the following strongly convex programming:

4 $$ \min \biggl\{ f(x)+\frac{\beta}{2}\Vert Ax-b \Vert ^{2}|Ax=b, x\in\mathcal{R}^{n} \biggr\} , $$

where the constant $\beta>0$ is a penalty parameter. Introducing the Lagrange multiplier $\lambda\in\mathcal{R}^{m}$ to the linear constraints $Ax=b$, we get the Lagrangian function associated with Problem (4):

$$ \mathcal{L}(x,\lambda)=f(x)+\lambda^{\top}(Ax-b)+ \frac{\beta}{2} \Vert Ax-b \Vert ^{2}, $$

which is also the augmented Lagrangian function associated with Problem (1). Then the dual function is denoted by

$$ G(\lambda)=\inf_{x}\mathcal{L}(x,\lambda), $$

and the dual problem of (4) is

$$ \max_{\lambda\in\mathcal{R}^{m}}G(\lambda). $$

Due to the strong convexity of the objective function of Problem (4), $G(\lambda)$ is continuously differentiable at any $\lambda\in\mathcal{R}^{m}$, and $\nabla G(\lambda)=-(Ax(\lambda)-b)$, where $x(\lambda)= \mathrm{argmin}_{x}\mathcal{L}(x,\lambda)$ (see, e.g., Theorem 1 of [9]). Solving the above dual problem by the gradient ascent method, we get a benchmark solver for Problem (1): the augmented Lagrangian method (ALM) [10, 11], which first minimizes the Lagrangian function of Problem (4) with respect to *x* by fixing $\lambda=\lambda^{k}$ to get $x(\lambda^{k})$, and set $x^{k+1}=x(\lambda^{k})$; then it updates the Lagrange multiplier *λ*. Specifically, for given $\lambda^{k}$, the *k*th iteration of PALM for Problem (1) reads

5 $$ \textstyle\begin{cases} x^{k+1}=\mathop{\arg\min}_{x\in\mathcal{R}^{n}} \{\mathcal{L}(x,\lambda ^{k}) \} , \\ \lambda^{k+1}=\lambda^{k}+\gamma\beta(Ax^{k+1}-b), \end{cases} $$

where $\gamma\in(0,2)$ is a relaxation factor. Though ALM plays a fundamental role in the algorithmic development of Problem (1), the cost of solving its first subproblem is often high for general $f(\cdot)$ and *A*. To address this issue, many proximal ALMs [3, 12–14] are developed by adding the proximal term $\frac{1}{2}\Vert x-x^{k} \Vert _{G}^{2}$ to the *x*-related subproblem, where $G\in\mathcal{R}^{n\times n}$ is a semi-definite matrix. By setting $G=\tau I_{n}-\beta A^{\top}A$ with $\tau>\beta \Vert A^{\top}A \Vert $, the *x*-related subproblem reduces to the following form:

$$ x^{k+1}=\mathop{\arg\min}_{x\in\mathcal{R}^{n}} \biggl\{ \begin{aligned} f(x)+ \frac{\tau}{2} \bigl\Vert x-u^{k} \bigr\Vert ^{2} \end{aligned} \biggr\} , $$

where $u^{k}=\frac{1}{\tau}(Gx^{k}-A^{\top}\lambda^{k}+\beta A ^{\top}b)$. The above subproblem is often simple enough to have a closed-form solution or can be easily solved up to a high precision. The proximal ALM is so instructive, and along this philosophy, a lot of efficient proximal ALM-type methods [3, 15–18] have been proposed. However, a new difficult problem has arisen for the proximal ALM-type methods, which is how to determine the optimal value of the proximal parameter *τ*. Numerical results indicate that smaller values of *τ* can often speed up the corresponding proximal ALM-type method [19].

In [20], Fazel et al. pointed out that the proximal matrix *G* should be as small as possible, while the subproblem related to the primal variable *x* is still relatively easy to tackle. Furthermore, though any $\tau>\beta \Vert A^{\top}A \Vert $ can ensure the global convergence of proximal ALM-type methods, the computation of the norm $\Vert A^{\top}A \Vert $ is often high for some problems in practice, especially for large *n*. Therefore, it is meaningful to relax the feasible region of the proximal parameter *τ*. Quite recently some efficient methods with relaxed proximal matrix [21–24] have been developed for Problem (1). Specifically, He et al. [23] proposed a positive-indefinite proximal augmented Lagrangian method, in which the proximal matrix is $G=G_{0}-(1-\alpha)\beta A^{\top}A$, where $G_{0}$ is an arbitrarily positive definite matrix in $\mathcal{R} ^{n}$, and $\alpha\in(\frac{2+\gamma}{4},1)$. If we set $G_{0}= \tau I_{n}-\alpha\beta A^{\top}A$ with $\tau>\alpha\beta \Vert A^{\top}A \Vert $, then $G=\tau I_{n}-\beta A^{\top}A$, which maybe indefinite.

Many research results on the convergence speed of the ALM-type methods have been presented recently. For the classical ALM, He et al. [25] firstly established the worst-case $\mathcal{O}(1/t)$ convergence rate in the non-ergodic sense, and further developed a new ALM-type method, which has $\mathcal{O}(1/t ^{2})$ convergence rate. However, for inequality (3.13) in [25], the authors only proved $L(x^{*},\lambda^{*})-L( \tilde{x}^{k},\tilde{\lambda}^{k})\leq C/(k+1)^{2}$ with $C>0$, and we cannot ensure $L(x^{*},\lambda^{*})-L(\tilde{x}^{k}, \tilde{\lambda}^{k})\geq0$. Similar problem occurs in Theorem 2.2 of [26]. In the following, we shall prove that the inequality $L(x^{*},\lambda ^{*})-L(\tilde{x}^{k},\tilde{\lambda}^{k})\geq0$ holds for the method in [26]. Quite recently, by introducing an ingeniously designed sequence $\{\theta_{k}\}$, Lu et al. [27] proposed a fast PALM-type method without proximal term, which has $\mathcal{O}(1/t^{2})$ convergence rate.

In this paper, based on the study of [27], we are going to further study the augmented Lagrangian method and develop a new fast proximal ALM-type method with indefinite proximal regularization, whose worst-case convergence rate is $\mathcal{O}(1/t^{2})$ in a non-ergodic sense. Furthermore, a relaxation factor $\gamma\in(0,2)$ is attached to the updated formula of our new method, which is often beneficial to speed up convergence in practice.

The rest of this paper is organized as follows. In Section 2, we list some necessary notations. We then give the proximal ALM with indefinite proximal regularization (PALM-IPR) and show its worst-case $\mathcal{O}(1/t^{2})$ convergence rate in Section 3. In Section 4, numerical experiments are conducted to illustrate the efficiency of PALM-IPR. Finally, some conclusions are drawn in Section 5.

## Preliminaries

In this section, we give some notations used in the following analysis and present two criteria to measure the worst-case $\mathcal{O}(1/t)$ convergence rate of PALM type methods. At the end of this section, we prove that the inequality $L(x^{*},\lambda ^{*})-L(\tilde{x}^{k},\tilde{\lambda}^{k})\geq0$ holds for the method in [26].

Throughout, we use the following standard notations. For any two vectors $x,y\in\mathcal{R}^{n}$, $\langle x,y\rangle$ or $x^{\top}y$ denote their inner products. The symbols $\Vert \cdot \Vert _{1}$ and $\Vert \cdot \Vert $ represent the $\ell_{1}$-norm and $\ell_{2}$-norm for vector variables, respectively. $I_{n}$ denotes the *n*-dimensional identity matrix. If the matrix $G\in\mathcal{R}^{n\times n}$ is symmetric, we use the symbol $\Vert x \Vert _{G}^{2}$ to denote $x^{\top}Gx$ even if *G* is indefinite; $G\succ0$ (resp., $G\succeq0$) denotes that the matrix *G* is positive definite (resp., semi-definite).

The following identity will be used in the following analysis:

6 $$ \langle x-y,x-z\rangle=\frac{1}{2} \bigl(\Vert x-y \Vert ^{2}+\Vert x-z \Vert ^{2}-\Vert y-z \Vert ^{2} \bigr), \quad x,y,z\in\mathcal{R}^{n}. $$

### Definition 2.1

A point $({x}^{*},\lambda^{*})$ is a Karush-Kuhn-Tucker (KKT) point of Problem (1) if the following conditions are satisfied:

7 $$ \textstyle\begin{cases} 0\in\partial f(x^{*})+A^{\top}\lambda^{*}, \\ Ax^{*}=b. \end{cases} $$

Note that the two conditions of (7) correspond to the dual feasibility and the primal feasibility of Problem (1), respectively. The solution set of KKT system (7), denoted by $\mathcal{W}^{*}$, is nonempty under the nonempty solution set of (1). By (7) and the property of the convex function $f(\cdot)$, for any $({x}^{*},\lambda^{*})\in\mathcal{W}^{*}$, we have

8 $$ f(x)-f \bigl(x^{*} \bigr)+ \bigl(x-x^{*} \bigr)^{\top} \bigl(A^{\top}\lambda^{*} \bigr)\geq0, \quad\forall x\in\mathcal{R}^{n}. $$

Based on (8) and $Ax^{*}=b$, we have the following proposition.

### Proposition 2.1

([28])

*Vector* $\tilde{x}\in \mathcal{R} ^{n}$ *is an optimal solution to Problem* (1) *if and only if there exists* $r>0$ *such that*

9 $$ f(\tilde{x})-f \bigl(x^{*} \bigr)+ \bigl( \tilde{x}-x^{*} \bigr)^{\top} \bigl(A^{\top} \lambda^{*} \bigr)+ \frac{r}{2}\Vert A\tilde{x}-b \Vert ^{2}=0, $$

*where* $(x^{*},\lambda^{*})\in\mathcal{W}^{*}$.

Now let us review two different criteria to measure the worst-case $\mathcal{O}(1/t^{2})$ convergence rate in [28, 29].

(1) In [29], Xu presented the following criterion:

10 $$ \textstyle\begin{cases} \vert f({x}^{t})-f(x^{*}) \vert \leq\frac{C}{(t+1)^{2}}, \\ \Vert A{x}^{t}-b \Vert \leq\frac{C}{\Vert \lambda^{*} \Vert (t+1)^{2}}, \end{cases} $$

where $C>0$. The second inequality of (10) implies that there must exist at least one $({x}^{*},\lambda^{*})\in\mathcal{W}^{*}$ with $\lambda^{*}\neq0$.

(2) In [28], Lin et al. proposed the following criterion:

11 $$ f \bigl(x^{t} \bigr)-f \bigl(x^{*} \bigr)+ \bigl(x^{t}-x^{*} \bigr)^{\top} \bigl(A^{\top} \lambda^{*} \bigr)+ \frac{c}{2} \bigl\Vert Ax^{t}-b \bigr\Vert ^{2}\leq\frac{C}{(t+1)^{2}}, $$

where $c, C>0$. Obviously, inequality (11) is motivated by equality (9). Compared with (10), the criterion (11) is more reasonable. Therefore, we shall use (11) to measure the $\mathcal{O}(1/t^{2})$ convergence rate of our new method.

Now, we prove that the inequality $L(x^{*},\lambda^{*})-L(\tilde{x}^{k},\tilde {\lambda}^{k})\geq0$ holds for the iteration method proposed in [26].

### Theorem 2.1

*For the KKT point* $(x^{*},\lambda^{*})$ *of Problem* (1), *the tuple* $(x^{k+1},\tilde{\lambda}^{k+1})$ *generated by the method in* [26] *satisfies*

$$ \mathcal{L}\bigl(x^{k+1},\tilde{\lambda}^{k+1}\bigr)\leq \mathcal{L}\bigl(x^{*},\lambda^{*}\bigr). $$

### Proof

Since $x^{k+1}$ is generated by the following subproblem:

$$x^{k+1}=\mathop{\arg\min}_{x\in\mathcal{R}^{n}}\biggl\{ f(x)+\frac{\beta }{2} \biggl\Vert Ax-b+\frac{\lambda^{k}}{\beta} \biggr\Vert ^{2}\biggr\} , $$

then it holds that

$$0\in\partial f\bigl(x^{k+1}\bigr)+A^{\top}\bigl( \lambda^{k}+\beta\bigl(Ax^{k+1}-b\bigr)\bigr), $$

i.e.,

$$-A^{\top}\tilde{\lambda}^{k+1}\in\partial f \bigl(x^{k+1}\bigr). $$

This and the convexity of the function $f(\cdot)$ yield

$$f\bigl(x^{*}\bigr)-f\bigl(x^{k+1}\bigr)\geq\bigl\langle -A^{\top}\tilde{\lambda }^{k+1},x^{*}-x^{k+1}\bigr\rangle . $$

So, it holds that

$$\begin{aligned} \mathcal{L} \bigl(x^{*},\lambda^{*} \bigr)&=f \bigl(x^{*} \bigr) \\ & \geq f \bigl(x^{k+1} \bigr)+ \bigl\langle -A^{\top}\tilde{ \lambda}^{k+1},x^{*}-x^{k+1} \bigr\rangle \\ & \geq f \bigl(x^{k+1} \bigr)+ \bigl\langle -\tilde{ \lambda}^{k+1},Ax^{*}-Ax^{k+1} \bigr\rangle \\ & \geq f \bigl(x^{k+1} \bigr)+ \bigl\langle \tilde{ \lambda}^{k+1},Ax^{k+1}-b \bigr\rangle \\ &=\mathcal{L} \bigl(x^{k+1},\tilde{\lambda}^{k+1} \bigr). \end{aligned}$$

This completes the proof. □

## PALM-IPR and its convergence rate

In this section, we first present the proximal ALM with indefinite proximal regularization (PALM-IPR) for Problem (1) and then prove its convergence rate step by step.

To present our new method, let us define a sequence $\{\theta_{k}\}$ which satisfies $\theta_{0}=1$, $\theta_{k}>0$ and

12 $$ \frac{1-\theta_{k+1}}{\theta_{k+1}^{2}}=\frac{1}{\theta_{k}^{2}}. $$

Then the sequence $\{\theta_{k}\}$ has the following properties [27]:

13 $$ \sum_{k=0}^{t} \frac{1}{\theta_{k}}=\frac{1}{\theta_{t}^{2}}, $$

and

14 $$ \theta_{k}\leq\frac{2}{k+2}, \quad\forall k\geq 0. $$

### Algorithm 3.1

(PALM-IPR for Problem (1))

*Step 1.* Input the parameters $\beta_{0}=\theta_{0}=1, \gamma \in(0,2)$, the tolerance $\varepsilon>0$. Initialize $x^{0}\in \mathcal{R}^{n}, z^{0}\in\mathcal{R}^{n}, \lambda^{0}\in\mathcal{R} ^{m}$ and $G_{0}\in\mathcal{R}^{n\times n}$. Set $k:=0$.

*Step 2.* Compute

15 $$\begin{aligned}& z^{k+1}=\mathop{\arg\min}_{x\in\mathcal{R}^{n}} \biggl\{ f(x)+ \frac{\beta_{k}}{2} \biggl\Vert Ax-b+\frac{\lambda^{k}}{\beta_{k}} \biggr\Vert ^{2}+\frac{ \beta_{k}}{2} \bigl\Vert x-z^{k} \bigr\Vert _{G_{k}}^{2} \biggr\} ; \end{aligned}$$

16 $$\begin{aligned}& x^{k+1}=(1-\theta_{k})x^{k}+ \theta_{k}z^{k+1}; \end{aligned}$$

17 $$\begin{aligned}& \lambda^{k+1}=\lambda^{k}+\gamma \beta_{k} \bigl(Az^{k+1}-b \bigr); \end{aligned}$$

18 $$\begin{aligned}& \theta_{k+1}=\frac{-\theta_{k}^{2}+\sqrt{\theta _{k}^{4}+4\theta_{k} ^{2}}}{2}; \end{aligned}$$

19 $$\begin{aligned}& \beta_{k+1}=\frac{1}{\theta_{k+1}}. \end{aligned}$$

*Step 3.* If $\Vert x^{k}-{x}^{k+1} \Vert +\Vert \lambda ^{k}-\lambda^{k+1} \Vert \leq\varepsilon$, then stop; otherwise, choose $G_{k+1}\in \mathcal{R}^{n\times n}$, and set $k:=k+1$. Go to Step 2.

To prove the global convergence of PALM-IPR, we need to impose some restrictions on the proximal matrix $G_{k}$, which is stated as follows.

### Assumption 3.1

The proximal matrix $G_{k}$ is set as $G_{k}= \theta_{k}^{2}(D_{k}-(1-\alpha)A^{\top}A)$, where $\alpha\in(\frac{2+ \gamma}{4},1)$ and the matrix $D_{k}\succeq0$, $\forall k\geq1$.

### Remark 3.1

The proximal matrix $G_{k}$ maybe indefinite. For example, if we set $D_{k}=0$, then $G_{k}=-(1-\alpha)\theta_{k}^{2}A ^{\top}A$, which is indefinite when *A* is full-column rank.

Using the first-order optimality condition of the subproblem of PALM-IPR, we can deduce the following one-iteration result.

### Lemma 3.1

*Let* $\{(x^{k},y^{k},z^{k},\lambda^{k})\} _{k\geq0}$ *be the sequence generated by PALM*-*IPR*. *For any* $x\in\mathcal{R}^{n}$, *it holds that*

20 $$\begin{aligned} &\frac{1-\theta_{k+1}}{\theta_{k+1}^{2}} \bigl(f \bigl(x^{k+1} \bigr)-f(x) \bigr)- \frac{1}{ \gamma\theta_{k}} \bigl\langle \lambda^{k+1}+(\gamma-1) \lambda^{k},Ax-Az ^{k+1} \bigr\rangle \\ &\quad\leq\frac{1-\theta_{k}}{\theta_{k}^{2}} \bigl(f \bigl(x^{k} \bigr)-f(x) \bigr)+ \frac{1}{2\theta _{k}^{2}} \bigl( \bigl\Vert z^{k}-x \bigr\Vert _{G_{k}}^{2}- \bigl\Vert z^{k+1}-x \bigr\Vert _{G_{k}}^{2}- \bigl\Vert z^{k+1}-z ^{k} \bigr\Vert _{G_{k}}^{2} \bigr). \end{aligned}$$

### Proof

From the first-order optimality condition for *z*-related subproblem (15), we have

21 $$\begin{aligned} 0&\in\partial f \bigl(z^{k+1} \bigr)+ \beta_{k} A^{\top} \biggl(Az^{k+1}-b+ \frac{1}{ \beta_{k}} \lambda^{k} \biggr)+\beta_{k}G_{k} \bigl(z^{k+1}-z^{k} \bigr) \\ & =\partial f \bigl(z^{k+1} \bigr)+A^{\top}\tilde{\lambda }^{k}+\beta_{k}G_{k} \bigl(z ^{k+1}-z^{k} \bigr), \end{aligned}$$

where $\tilde{\lambda}^{k}=\lambda^{k}+\beta_{k}(Az^{k+1}-b)$. Then, from the convexity of $f(\cdot)$ and (21), we have

22 $$ f(x)-f \bigl(z^{k+1} \bigr)\geq- \bigl\langle A^{\top}\tilde{\lambda}^{k}+\beta_{k}G _{k} \bigl(z^{k+1}-z^{k} \bigr),x-z^{k+1} \bigr\rangle . $$

From (16) and the convexity of $f(\cdot)$ again, we thus get

$$\begin{aligned} f \bigl(x^{k+1} \bigr) &\leq(1-\theta_{k})f \bigl(x^{k} \bigr)+\theta_{k}f \bigl(z^{k+1} \bigr) \\ &\leq(1-\theta_{k})f \bigl(x^{k} \bigr)+ \theta_{k} \bigl(f(x)+ \bigl\langle A^{\top} \tilde{\lambda }^{k}+\beta_{k}G_{k} \bigl(z^{k+1}-z^{k} \bigr),x-z^{k+1} \bigr\rangle \bigr), \end{aligned}$$

where the second inequality follows from (22). Then, by rearranging terms of the above inequality, we arrive at

$$\begin{aligned} &f \bigl(x^{k+1} \bigr)-f(x)-\theta_{k} \bigl\langle A^{\top}\tilde{\lambda}^{k},x-z ^{k+1} \bigr\rangle \\ &\quad\leq(1-\theta_{k}) \bigl(f \bigl(x^{k} \bigr)-f(x) \bigr)+ \bigl\langle G_{k} \bigl(z^{k+1}-z^{k} \bigr),x-z ^{k+1} \bigr\rangle \\ &\quad\leq(1-\theta_{k}) \bigl(f \bigl(x^{k} \bigr)-f(x) \bigr)+ \frac{1}{2} \bigl( \bigl\Vert z^{k}-x \bigr\Vert _{G_{k}} ^{2}- \bigl\Vert z^{k+1}-x \bigr\Vert _{G_{k}}^{2}- \bigl\Vert z^{k+1}-z^{k} \bigr\Vert _{G_{k}}^{2} \bigr), \end{aligned}$$

where the second inequality uses (19), and the third inequality comes from identity (6). Dividing both sides of the above inequality by $\theta_{k}^{2}$, we get

$$\begin{aligned} &\frac{1}{\theta_{k}^{2}}f \bigl(x^{k+1} \bigr)-f(x)-\frac{1}{\theta _{k}} \bigl\langle A^{\top}\tilde{\lambda}^{k},x-z^{k+1} \bigr\rangle \\ &\quad\leq\frac{1-\theta_{k}}{\theta_{k}^{2}} \bigl(f \bigl(x^{k} \bigr)-f(x) \bigr)+ \frac{1}{2 \theta_{k}^{2}} \bigl( \bigl\Vert z^{k}-x \bigr\Vert _{G_{k}}^{2}- \bigl\Vert z^{k+1}-x \bigr\Vert _{G_{k}}^{2}- \bigl\Vert z^{k+1}-z^{k} \bigr\Vert _{G_{k}}^{2} \bigr). \end{aligned}$$

From (17), we have

$$\begin{aligned} &\tilde{\lambda}^{k}=\lambda^{k}+\frac{\lambda^{k+1}-\lambda^{k}}{ \gamma}= \frac{\lambda^{k+1}+(\gamma-1)\lambda^{k}}{\gamma}. \end{aligned}$$

Substituting this into the above inequality and using (12) lead to (20). This completes the proof. □

### Lemma 3.2

*Let* $\{(x^{k},y^{k},z^{k},\lambda^{k})\} _{k\geq0}$ *be the sequence generated by PALM*-*IPR*. *For any* $(x,\lambda)\in \mathcal{R}^{m+n}$ *with* $Ax=b$, *it holds that*

23 $$\begin{aligned} &\frac{1-\theta_{k+1}}{\theta_{k+1}^{2}} \bigl(f \bigl(x^{k+1} \bigr)-f(x) \bigr)-\frac{1- \theta_{k}}{\theta_{k}^{2}} \bigl(f \bigl(x^{k} \bigr)-f(x) \bigr)+ \frac{1}{\theta_{k}} \bigl\langle \lambda,Az^{k+1}-b \bigr\rangle \\ &\quad\leq\frac{1}{2\theta_{k}^{2}} \bigl( \bigl\Vert z^{k}-x \bigr\Vert _{G_{k}}^{2}- \bigl\Vert z^{k+1}-x \bigr\Vert _{G _{k}}^{2}- \bigl\Vert z^{k+1}-z^{k} \bigr\Vert _{G_{k}}^{2} \bigr) \\ &\quad\quad{}+\frac{1}{2\gamma^{2}} \bigl[\gamma \bigl( \bigl\Vert \lambda^{k}-\lambda \bigr\Vert ^{2}- \bigl\Vert \lambda^{k+1}-\lambda \bigr\Vert ^{2} \bigr)-(2-\gamma) \bigl\Vert \lambda^{k+1}-\lambda^{k} \bigr\Vert ^{2} \bigr]. \end{aligned}$$

### Proof

Adding the term $\frac{1}{\theta_{k}}\langle\lambda,Az ^{k+1}-b\rangle$ to both sides of (20), we get

24 $$\begin{aligned} &\frac{1-\theta_{k+1}}{\theta_{k+1}^{2}} \bigl(f \bigl(x^{k+1} \bigr)-f(x) \bigr)-\frac{1- \theta_{k}}{\theta_{k}^{2}} \bigl(f \bigl(x^{k} \bigr)-f(x) \bigr)+ \frac{1}{\theta_{k}} \bigl\langle \lambda,Az^{k+1}-b \bigr\rangle \\ &\quad \leq\frac{1}{2\theta_{k}^{2}} \bigl( \bigl\Vert z^{k}-x \bigr\Vert _{G_{k}}^{2}- \bigl\Vert z^{k+1}-x \bigr\Vert _{G _{k}}^{2}- \bigl\Vert z^{k+1}-z^{k} \bigr\Vert _{G_{k}}^{2} \bigr) \\ &\quad\quad{}+\frac{1}{\theta_{k}} \biggl\langle \lambda-\frac{\lambda ^{k+1}+(\gamma-1) \lambda^{k}}{\gamma},Az^{k+1}-b \biggr\rangle . \end{aligned}$$

Now, let us deal with the term $\langle\lambda-\frac{\lambda^{k+1}+( \gamma-1)\lambda^{k}}{\gamma},Az^{k+1}-b\rangle$. By (17), we have

$$\begin{aligned} & \biggl\langle \lambda-\frac{\lambda^{k+1}+(\gamma-1)\lambda ^{k}}{\gamma },Az^{k+1}-b \biggr\rangle \\ &\quad=\frac{1}{\gamma^{2}\beta_{k}} \bigl\langle \gamma\lambda -\lambda^{k+1}-( \gamma-1)\lambda^{k},\lambda^{k+1}-\lambda^{k} \bigr\rangle \\ &\quad=\frac{1}{\gamma^{2}\beta_{k}} \bigl(\gamma \bigl\langle \lambda- \lambda^{k}, \lambda^{k+1}-\lambda^{k} \bigr\rangle - \bigl\Vert \lambda^{k+1}-\lambda^{k} \bigr\Vert ^{2} \bigr) \\ &\quad=\frac{1}{2\gamma^{2}\beta_{k}} \bigl[\gamma \bigl( \bigl\Vert \lambda^{k}- \lambda \bigr\Vert ^{2}- \bigl\Vert \lambda^{k+1}-\lambda \bigr\Vert ^{2} \bigr)-(2-\gamma) \bigl\Vert \lambda^{k+1}- \lambda^{k} \bigr\Vert ^{2} \bigr]. \end{aligned}$$

Substituting the above equality into (24) and using (19), we get (23) immediately. This completes the proof. □

Let us further deal with the term $\Vert z^{k}-x \Vert _{G_{k}}^{2}-\Vert z^{k+1}-x \Vert _{G_{k}}^{2}-\Vert z^{k+1}-z^{k} \Vert _{G_{k}}^{2}$ on the right-hand side of (24).

### Lemma 3.3

*Let* $\{(x^{k},y^{k},z^{k},\lambda^{k})\} _{k\geq0}$ *be the sequence generated by PALM*-*IPR*. *For any* $(x,\lambda)\in \mathcal{R}^{m+n}$ *with* $Ax=b$, *it holds that*

25 $$\begin{aligned} &\frac{1}{\theta_{k}^{2}} \bigl( \bigl\Vert z^{k}-x \bigr\Vert _{G_{k}}^{2}- \bigl\Vert z^{k+1}-x \bigr\Vert _{G _{k}}^{2}- \bigl\Vert z^{k+1}-z^{k} \bigr\Vert _{G_{k}}^{2} \bigr) \\ &\quad\leq \bigl\Vert z^{k}-x \bigr\Vert _{D_{k}}^{2}- \bigl\Vert z^{k+1}-x \bigr\Vert _{D_{k}}^{2}- \bigl\Vert z^{k+1}-z ^{k} \bigr\Vert _{D_{k}}^{2} \\ &\quad\quad{}+(1-\alpha) \bigl( \bigl\Vert Az^{k}-b \bigr\Vert ^{2}- \bigl\Vert Az^{k+1}-b \bigr\Vert ^{2} \bigr)+4(1-\alpha) \bigl\Vert Az^{k+1}-b \bigr\Vert ^{2}. \end{aligned}$$

### Proof

By Assumption 3.1, we have

$$\begin{aligned} &\frac{1}{\theta_{k}^{2}} \bigl( \bigl\Vert z^{k}-x \bigr\Vert _{G_{k}}^{2}- \bigl\Vert z^{k+1}-x \bigr\Vert _{G _{k}}^{2}- \bigl\Vert z^{k+1}-z^{k} \bigr\Vert _{G_{k}}^{2} \bigr) \\ &\quad= \bigl\Vert z^{k}-x \bigr\Vert _{D_{k}}^{2}- \bigl\Vert z^{k+1}-x \bigr\Vert _{D_{k}}^{2}- \bigl\Vert z^{k+1}-z^{k} \bigr\Vert _{D_{k}}^{2} \\ &\quad \quad{} +(1-\alpha) \bigl(- \bigl\Vert Az^{k}-Ax \bigr\Vert ^{2}+ \bigl\Vert Az^{k+1}-Ax \bigr\Vert ^{2}+ \bigl\Vert Az^{k}-Az^{k+1} \bigr\Vert ^{2} \bigr) \\ &\quad= \bigl\Vert z^{k}-x \bigr\Vert _{D_{k}}^{2}- \bigl\Vert z^{k+1}-x \bigr\Vert _{D_{k}}^{2}- \bigl\Vert z^{k+1}-z^{k} \bigr\Vert _{D_{k}}^{2} \\ &\quad \quad{} +(1-\alpha) \bigl(- \bigl\Vert Az^{k}-b \bigr\Vert ^{2}+ \bigl\Vert Az^{k+1}-b \bigr\Vert ^{2}+ \bigl\Vert \bigl(Az^{k}-b \bigr)- \bigl(Az^{k+1}-b \bigr) \bigr\Vert ^{2} \bigr). \end{aligned}$$

Using the inequality $\Vert \xi-\eta \Vert ^{2}\leq2\Vert \xi \Vert ^{2}+2\Vert \eta \Vert ^{2}$ with $\xi=Az^{k}-b$ and $\eta=Az^{k+1}-b$, we get

$$ \bigl\Vert \bigl(Az^{k}-b \bigr)- \bigl(Az^{k+1}-b \bigr) \bigr\Vert ^{2}\leq2 \bigl\Vert Az^{k}-b \bigr\Vert ^{2}+2 \bigl\Vert Az^{k+1}-b \bigr\Vert ^{2}. $$

Substituting this inequality into the right-hand side of the above equality, we obtain assertion (25) immediately. This completes the proof. □

Then, from (23) and (25), we have

26 $$\begin{aligned}& \frac{1-\theta_{k+1}}{\theta_{k+1}^{2}} \bigl(f \bigl(x^{k+1} \bigr)-f(x) \bigr)- \frac{1- \theta_{k}}{\theta_{k}^{2}} \bigl(f \bigl(x^{k} \bigr)-f(x) \bigr)+ \frac{1}{\theta_{k}} \bigl\langle \lambda,Az^{k+1}-b \bigr\rangle \\& \quad\leq\frac{1}{2} \bigl[ \bigl\Vert z^{k}-x \bigr\Vert _{D_{k}}^{2}- \bigl\Vert z^{k+1}-x \bigr\Vert _{D_{k}}^{2}- \bigl\Vert z^{k+1}-z^{k} \bigl\Vert _{D_{k}}^{2}+(1-\alpha) \bigl( \bigr\Vert Az^{k}-b \bigl\Vert ^{2}- \bigr\Vert Az^{k+1}-b \bigr\Vert ^{2} \bigr) \bigr] \\& \quad\quad{}+4(1-\alpha) \bigl\Vert Az^{k+1}-b \bigr\Vert ^{2} \\& \quad\quad{}+\frac{1}{2\gamma^{2}} \bigl[\gamma \bigl( \bigl\Vert \lambda^{k}-\lambda \bigr\Vert ^{2}- \bigl\Vert \lambda^{k+1}-\lambda \bigr\Vert ^{2} \bigr)-(2-\gamma) \bigl\Vert \lambda^{k+1}-\lambda^{k} \bigr\Vert ^{2} \bigr] \\& \quad\leq\frac{1}{2\theta_{k}^{2}} \bigl[ \bigl\Vert z^{k}-x \bigr\Vert _{D_{k}}^{2}- \bigl\Vert z^{k+1}-x \bigr\Vert _{D_{k}}^{2}- \bigl\Vert z^{k+1}-z^{k} \bigr\Vert _{D_{k}}^{2} \\& \quad\quad{}+(1-\alpha) \bigl( \bigl\Vert Az^{k}-b \bigr\Vert ^{2}- \bigl\Vert Az^{k+1}-b \bigr\Vert ^{2} \bigr) \bigr] \\& \quad\quad{}+\frac{1}{2\gamma} \bigl( \bigl\Vert \lambda^{k}-\lambda \bigr\Vert ^{2}- \bigl\Vert \lambda^{k+1}-\lambda\bigr\Vert ^{2} \bigr)+\frac{2+\gamma-4\alpha}{2\gamma^{2}} \bigl\Vert \lambda^{k+1}-\lambda^{k} \bigr\Vert ^{2}, \end{aligned}$$

where the second inequality comes from (17) and the fact $\beta_{k}\geq1$.

Based on (26), we are going to prove the worst-case $\mathcal{O}(1/t^{2})$ convergence rate of PALM-IPR in an ergodic sense.

### Theorem 3.1

*Let* $\{(x^{k},y^{k},z^{k},\lambda^{k})\} _{k\geq0}$ *be the sequence generated by PALM*-*IPR*. *Then*

$$\begin{aligned} &f \bigl(x^{t+1} \bigr)-f \bigl(x^{*} \bigr)+ \bigl\langle A^{\top}\lambda^{*},x^{t+1}-x^{*} \bigr\rangle +\frac{4\alpha-2-\gamma}{2} \bigl\Vert Ax^{t+1}-b \bigr\Vert ^{2} \\ & \quad\leq \frac{2}{(t+2)^{2}} \bigl( \bigl\Vert z^{0}-x^{*} \bigr\Vert _{D_{0}}^{2}+(1- \alpha) \bigl\Vert Az^{0}-b \bigr\Vert ^{2} \bigr)+ \frac{2}{\gamma(t+2)^{2}} \bigl\Vert \lambda^{0}-\lambda^{*} \bigr\Vert ^{2}. \end{aligned}$$

### Proof

Setting $x=x^{*}$ and $\lambda=\lambda^{*}$ in (26), we get

$$\begin{aligned} & \frac{1-\theta_{k+1}}{\theta_{k+1}^{2}} \bigl(f \bigl(x^{k+1} \bigr)-f \bigl(x^{*} \bigr) \bigr)-\frac{1- \theta_{k}}{\theta_{k}^{2}} \bigl(f \bigl(x^{k} \bigr)-f \bigl(x^{*} \bigr) \bigr)+ \frac{1}{\theta_{k}} \bigl\langle \lambda^{*},Az^{k+1}-b \bigr\rangle \\ &\quad\leq \frac{1}{2} \bigl[ \bigl\Vert z^{k}-x^{*} \bigr\Vert _{D_{k}}^{2}- \bigl\Vert z^{k+1}-x^{*} \bigr\Vert _{D_{k}}^{2}- \bigl\Vert z^{k+1}-z^{k} \bigr\Vert _{D_{k}}^{2} \\ &\quad\quad{}+(1- \alpha) \bigl( \bigl\Vert Az^{k}-b \bigr\Vert ^{2}- \bigl\Vert Az^{k+1}-b \bigr\Vert ^{2} \bigr) \bigr] \\ &\quad\quad{}+\frac{1}{2\gamma} \bigl( \bigl\Vert \lambda^{k}- \lambda^{*} \bigr\Vert ^{2}- \bigl\Vert \lambda^{k+1}-\lambda^{*} \bigr\Vert ^{2} \bigr)+\frac{2+\gamma-4\alpha}{2\gamma^{2}} \bigl\Vert \lambda^{k+1}- \lambda^{k}\bigr\Vert ^{2}. \end{aligned}$$

Summing the above inequality over $k=1, 2, \ldots, t$ and by (17), we have

27 $$\begin{aligned} &\frac{1-\theta_{t+1}}{\theta_{t+1}^{2}}\bigl(f\bigl(x^{t+1}\bigr)-f \bigl(x^{*}\bigr)\bigr)+\sum_{k=0} ^{t}\frac{1}{\theta_{k}}\bigl\langle \lambda^{*},Az^{k+1}-b \bigr\rangle \\ &\quad\leq \frac{1}{2}\bigl(\bigl\Vert z^{0}-x^{*} \bigr\Vert _{D_{0}}^{2}+(1-\alpha )\bigl\Vert Az^{0}-b \bigr\Vert ^{2}\bigr)+\frac{1}{2 \gamma}\bigl\Vert \lambda^{0}-\lambda^{*} \bigr\Vert ^{2} \\ &\quad\quad{}+\frac{2+\gamma-4\alpha}{2\theta_{k}^{2}}\sum_{k=0}^{t} \bigl\Vert Az^{k+1}-b \bigr\Vert ^{2} \\ &\quad\leq \frac{1}{2}\bigl(\bigl\Vert z^{0}-x^{*} \bigr\Vert _{D_{0}}^{2}+(1-\alpha )\bigl\Vert Az^{0}-b \bigr\Vert ^{2}\bigr)+\frac{1}{2 \gamma}\bigl\Vert \lambda^{0}-\lambda^{*} \bigr\Vert ^{2} \\ &\quad\quad{}+\frac{2+\gamma-4\alpha}{2\theta_{k}}\sum_{k=0}^{t} \bigl\Vert Az^{k+1}-b \bigr\Vert ^{2}, \end{aligned}$$

where the second inequality follows from $\theta_{k}\leq1$ and $\alpha \in(\frac{2+\gamma}{4},1)$. By equations (65), (68) of [27], we have

$$ \sum_{k=0}^{t}\frac{z^{k+1}}{\theta_{k}}= \frac{1}{\theta_{t}^{2}}x ^{t+1}, $$

and

$$ \sum_{k=0}^{t}\frac{1}{\theta_{k}} \bigl\Vert Az^{k+1}-b \bigr\Vert ^{2}\geq\frac{1}{ \theta_{t}^{2}} \bigl\Vert Ax^{t+1}-b \bigr\Vert ^{2}. $$

Substituting the above two relationships into (27) and using (13), we get

$$\begin{aligned} & \frac{1-\theta_{t+1}}{\theta_{t+1}^{2}} \bigl(f \bigl(x^{t+1} \bigr)-f \bigl(x^{*} \bigr) \bigr)+\frac{1}{ \theta_{t}^{2}} \bigl\langle \lambda^{*},Ax^{t+1}-b \bigr\rangle +\frac{4\alpha-2- \gamma}{2\theta_{t}^{2}} \bigl\Vert Ax^{t+1}-b \bigr\Vert ^{2} \\ &\quad \leq \frac{1}{2} \bigl( \bigl\Vert z^{0}-x^{*} \bigr\Vert _{D_{0}}^{2}+(1- \alpha) \bigl\Vert Az^{0}-b \bigr\Vert ^{2} \bigr)+ \frac{1}{2 \gamma} \bigl\Vert \lambda^{0}-\lambda^{*} \bigr\Vert ^{2}. \end{aligned}$$

Then multiplying two sides of the above inequality by $\theta_{t}^{2}$ and using (12) lead to

$$\begin{aligned} &f \bigl(x^{t+1} \bigr)-f \bigl(x^{*} \bigr)+ \bigl\langle \lambda^{*},Ax^{t+1}-b \bigr\rangle + \frac{4 \alpha-2-\gamma}{2} \bigl\Vert Ax^{t+1}-b \bigr\Vert ^{2} \\ &\quad \leq \frac{\theta_{t}^{2}}{2} \bigl( \bigl\Vert z^{0}-x^{*} \bigr\Vert _{D_{0}}^{2}+(1- \alpha) \bigl\Vert Az^{0}-b \bigr\Vert ^{2} \bigr)+ \frac{\theta_{t}^{2}}{2\gamma} \bigl\Vert \lambda^{0}-\lambda^{*} \bigr\Vert ^{2} \\ &\quad \leq \frac{2}{(t+2)^{2}} \bigl( \bigl\Vert z^{0}-x^{*} \bigr\Vert _{D_{0}}^{2}+(1- \alpha) \bigl\Vert Az^{0}-b \bigr\Vert ^{2} \bigr)+ \frac{2}{\gamma(t+2)^{2}} \bigl\Vert \lambda^{0}-\lambda^{*} \bigr\Vert ^{2} , \end{aligned}$$

where the second inequality follows from (14). The above inequality with $Ax^{*}=b$ results in the conclusion of the theorem. The proof is completed. □

## Numerical results

In this section, we apply PALM-IPR to some practical applications and report the numerical results. All the codes were written by Matlab R2010a and conducted on ThinkPad notebook with 2GB of memory.

### Problem 4.1

(Quadratic programming)

Firstly, let us test PALM-IPR on equality constrained quadratic programming (ECQP) [29] to validate its stability:

$$\min F(x)=\frac{1}{2}x^{\top}Qx+c^{\top}x, \quad \textrm{s.t. } Ax=b. $$

We set the problem size to $m=20, n=100$ and generate $A\in\mathcal {R}^{m\times n}, b, c$ and $Q\in\mathcal{R}^{n\times n}$ according to the standard Gaussian distribution. We compare PALM-IPR with the classical ALM with $\beta=1$. For PALM-IPR, we set $G_{k}=0$ for simplicity. We have tested the experiment sixty times, and the numerical results are listed in Table 1, in which ‘NC’ means the number of convergence; ‘ND’ means the number of divergence and ‘Ratio’ means the ratio of succession. From Table 1, we can see that PALM-IPR performs much more stably than the classical ALM.

**Table 1:** **Comparison of PALM-IPR with ALM**

**Method**	**NC**	**ND**	**Ratio**
PALM-IPR	59	1	98.33%
ALM	36	24	60.00%

### Problem 4.2

(Compressive sensing: the linearly constrained $\ell_{1}-\ell_{2}$ minimization problem (2))

Now, let us test PALM-IPR on the compressive sensing to validate its acceleration. For this problem, we firstly elaborate on how to solve subproblem (15) resulting from PALM-IPR. Due to the existence of the augmented term $\Vert Ax-b \Vert ^{2}$, we cannot get the closed-form solution if we do not attach the proximal term $\frac{1}{2}\Vert x-z^{k} \Vert _{G_{k}}^{2}$. In this case, we have to solve the subproblem inexactly, which is often time-consuming. Therefore, we choose the proximal matrix $G_{k}$ in (15) as $G_{k}=\theta_{k}^{2}(D _{k}-(1-\alpha)A^{\top}A)$, in which $D_{k}=\frac{1}{\beta_{k}\theta _{k}^{2}}P_{k}+(1-\alpha)A^{\top}A$, $P_{k}=\tau_{k} I_{n}-\beta _{k} A^{\top}A$ and $\tau_{k}>\beta_{k}\Vert A^{\top}A \Vert $, and subproblem (15) can be written as

$$ {z}^{k+1}=\mathop{\arg\min}_{x\in\mathcal{R}^{n}} \biggl\{ f(x)+ \frac{\beta _{k}}{2} \biggl\Vert Ax-b+\frac{\lambda^{k}}{\beta_{k}} \biggr\Vert ^{2}+ \frac{1}{2} \bigl\Vert x-z^{k} \bigr\Vert _{P_{k}}^{2} \biggr\} , $$

which is equivalent to

$$ {z}^{k+1}=\mathop{\arg\min}_{x\in\mathcal{R}^{n}} \biggl\{ \begin{aligned} \Vert x \Vert _{1}+\frac{1+\mu\tau_{k}}{2\mu} \biggl\Vert \begin{aligned}x-\frac{\mu}{1+\mu\tau_{k}} \bigl(P_{k}z^{k}-A^{\top}\lambda^{k}+\beta_{k} A^{\top}b \bigr) \end{aligned} \biggr\Vert ^{2} \end{aligned} \biggr\} , $$

and has a closed-form solution as follows:

$$ {z}^{k+1}=\mathrm{shrink}_{1,2} \bigl(a^{k}, \mu/(1+ \mu\tau_{k}) \bigr)\doteq\operatorname{sign} \bigl(a^{k} \bigr) \cdot\max \bigl\{ 0, \bigl\vert a^{k} \bigr\vert -\mu/(1+\mu \tau_{k}) \bigr\} , $$

in which $a^{k}=\frac{\mu}{1+\mu\tau_{k}}(P_{k}z^{k}-A^{\top}\lambda^{k}+\beta _{k}A^{\top}b)$. Note that all computations are component-wise.

In this experiment, we set $m=\operatorname{\mathtt{floor}}(a\times n)$ and $k=\operatorname{\mathtt{floor}}(b\times m)$ with $n\in\{500,1\text{,}000, 1\text{,}500, 2\text{,}000,3\text{,}000\}$, where *k* is the number of random nonzero elements contained in the original signal. The sensing matrix *A* is generated by the following Matlab scripts: $\bar{A}=\operatorname{\mathtt{randn}}(m,n)$, $[Q, R] = \operatorname{\mathtt{qr}}(\bar{A}',0); A = Q'$, and the nonzero entries of the true signal $x^{*}$, whose values are sampled from the standard Gaussian: $x^{*}=\operatorname{\mathtt{zeros}}(n,1)$; $p=\operatorname{\mathtt{randperm}}(n)$; $x^{*}(p(1:k))= \operatorname{\mathtt{randn}}(k,1)$, are selected at random. The observed signal *b* is generated by $b=R'\setminus Ax^{*}$. In addition, we set $\mu=500$, $\gamma=1.3$. In this experiment, we set the proximal parameter $\tau_{k}=4\beta_{k}\Vert A^{\top}A \Vert $. Furthermore, the stopping criterion is

$$ \operatorname{RelErr}=\frac{\Vert x^{k}-x^{*} \Vert }{\Vert x^{*} \Vert }\leq5\%, $$

or the number of iterations exceeds 10^4^, where $x^{k}$ is the iterate generated by PALM-IPR. Furthermore, all initial points are set as $x^{0}=A^{\top}b, \lambda^{0}=0$. For comparison, we also give the numerical results of PALM-SDPR [14] and the proximal PALM with positive-indefinite proximal regularization (PALM-PIPR) [21], and the proximal matrix is set to be $G=\tau I_{n}- \beta A^{\top}A$ in PALM-SDPR and $G=0.9\tau I_{n}-\beta A^{\top}A$ in PALM-PIPR, and $\tau=1.1\beta \Vert A^{\top}A \Vert $, $\beta=2 \operatorname{\mathtt{mean}}(\mathtt{abs}(b))$. Furthermore, we set $\gamma=1$ in PALM-PIPR. Since the computational load of all three methods are almost the same at each iteration, we only list the number of iterations (‘Iter’), the relative error (‘RelErr’) when three methods achieve the stopping criterion. The numerical results are listed in Table 1, and in the view of statistics, all the results are the average of 10 runs for each pair $(n,a,b)$.

Numerical results in Table 2 indicate that: (1) All methods have succeeded in solving Problem (2) for all the scenarios; (2) The new method PALM-IPR outperforms PALM-SDPR and PALM-PIPR by taking a fewer number of iterations to converge except $(n,a,b)=(1\text{,}000,0.2,0.2)$.

**Table 2:** **Comparison of PALM-IPR with PALM-SDPR and PALM-PIPR**

***n***	***a***	***b***	**IPR**		**SDPR**		**PIPR**	
			**Iter**	**RelErr**	**Iter**	**RelErr**	**Iter**	**RelErr**
500	0.2	0.2	62.3	0.0488	93.2	0.0473	98.6	0.0491
	0.2	0.1	20.5	0.0482	73.6	0.0440	69.2	0.0443
1,000	0.2	0.2	91.9	0.0492	80.0	0.0476	84.3	0.0484
	0.2	0.1	20.6	0.0462	83.3	0.0469	79.9	0.0450
1,500	0.2	0.2	66.4	0.0493	77.5	0.0482	87.5	0.0485
	0.2	0.1	20.3	0.0471	76.6	0.0459	75.9	0.0453
2,000	0.2	0.2	44.7	0.0492	58.3	0.0491	54.0	0.0488
	0.2	0.1	19.1	0.0474	85.2	0.0481	86.5	0.0459
3,000	0.2	0.2	46.3	0.0488	86.8	0.0480	93.2	0.0477
	0.2	0.1	20.2	0.0474	69.3	0.0472	64.6	0.0496

## Conclusions

In this paper, an accelerated augmented proximal Lagrangian method with indefinite proximal regularization (PALM-IPR) for linearly constrained convex programming is proposed. Under mild conditions, we have established the worst-case $\mathcal{O}(1/t^{2})$ convergence rate in a non-ergodic sense of PALM-IPR. Two sets of numerical results, which illustrate that PALM-IPR performs better than some state-of-the-art solvers, are given.

Similar to our proposed method, the methods in [26, 30] also have the worst-case $\mathcal{O}(1/t^{2})$ convergence rate in a non-ergodic sense, but they often have to solve a difficult subproblem at each iteration, and some inner iteration has to be executed. A prominent characteristic of the methods in [26, 30] is that the parameter *β* can be any positive constant, but the parameter *β* in PALM-IPR changes with respect to the iteration counter *k*, and it can actually go to infinity as $k\rightarrow\infty$. In practice, we often observe that larger *β* usually induces to slower convergence. Therefore, the method with proximal term, faster convergence rate and constant parameter *β* deserves further research.
